# Supplementary material for: MAGqual: a stand-alone pipeline to assess the quality of metagenome-assembled genomes
Source: Microbiome. 2024 Nov 4;12:226. doi: 10.1186/s40168-024-01949-z (PMC11533350; doi:10.1186/s40168-024-01949-z)
Supplement: Supplementary file 2 — Additional file 1: Supplementary figures: Fig. 1: Total number of bases and contigs binned for CONCOCT, DAS_tool, BinSanity, MetaWRAP, MetaBAT2. Figure 2: Distribution of the completeness and contamination of CONCOCT, DAS_tool, BinSanity, MetaWRAP, MetaBAT2. Figure 3: Distribution of the the N50 lengths (bp) of the bins generated by CONCOCT, DAS_tool, BinSanity, MetaWRAP, MetaBAT2. Supplementary table: Table 1: Overall MAG quality of CONCOCT, DAS_tool, BinSanity, MetaWRAP, MetaBAT2 and total number of MAGs generated by each method [file 40168_2024_1949_MOESM1_ESM.html]

Example report from MAGqual: A standalone pipeline to assess the quality of metagenome-assembled genomes


# Example report from MAGqual: A standalone pipeline to assess the quality of metagenome-assembled genomes

#### Annabel Cansdale and James P.J. Chong

> #### Note: Report input files
>
> This report has been generated using the below files, if you are missing
> any samples please add the summary statistics CSV into the
> `./analysis/` directory and re-run this script.
>
> Files:
>
> ```
> ## [1] "analysis/CONCOCT_mag_qual_statistics.csv"  
> ## [2] "analysis/DAS_tool_mag_qual_statistics.csv" 
> ## [3] "analysis/BinSanity_mag_qual_statistics.csv"
> ## [4] "analysis/MetaWRAP_mag_qual_statistics.csv" 
> ## [5] "analysis/MetaBAT2_mag_qual_statistics.csv"
> ```

## Overall bin quality

**Table 1: Overall MAG quality of CONCOCT, DAS\_tool,
BinSanity, MetaWRAP, MetaBAT2 and total number of MAGs generated by each
method.**

## Binning comparison

**Figure 1: Total number of bases and contigs binned
for CONCOCT, DAS\_tool, BinSanity, MetaWRAP, MetaBAT2**

**Figure 2: Distribution of the completeness and
contamination of CONCOCT, DAS\_tool, BinSanity, MetaWRAP, MetaBAT2.**

### Additional plots

#### N50 length

**Figure 3: Distribution of the the N50 lengths (bp)
of the bins generated by CONCOCT, DAS\_tool, BinSanity, MetaWRAP,
MetaBAT2.**

#### Total length

**Figure 4: Distribution of the the length (bp) of
the bins generated by CONCOCT, DAS\_tool, BinSanity, MetaWRAP, MetaBAT2.**

#### Number of tRNAs encoded

**Figure 5: Distribution of the the number of unique
tRNA sequences (/20) extracted in the bins generated by CONCOCT,
DAS\_tool, BinSanity, MetaWRAP, MetaBAT2.**

## Metadata tables

### CONCOCT

### DAS\_tool

### BinSanity

### MetaWRAP

### MetaBAT2
